# Mechanical Properties and Tensile Model of Hemp-Fiber-Reinforced Poly(butylene adipate-*co*-terephthalate) Composite

**DOI:** 10.3390/ma15072445

**Published:** 2022-03-26

**Authors:** Deyong Zeng, Liang Zhang, Shaojin Jin, Youyuan Zhang, Cuicui Xu, Kai Zhou, Weihong Lu

**Affiliations:** 1 School of Medicine and Health, Harbin Institute of Technology, Harbin 150001, China; 18b925086@stu.hit.edu.cn (D.Z.); 19s025072@stu.hit.edu.cn (L.Z.); 2 National and Local Joint Engineering Laboratory for Synthesis, Transformation and Separation of Extreme Environmental Nutrients, Harbin 150001, China; 3 Shandong Hagong Biological Technology Co., Ltd., Jinan 250200, China; jinsj@hitrobotgroup.com (S.J.); zhangyouyuan@hitrobotgroup.com (Y.Z.); xucc@hitrobotgroup.com (C.X.); zhouk@hitrobotgroup.com (K.Z.)

**Keywords:** hemp fiber, poly(butylene adipate-*co*-terephthalate), mechanical properties, stretch model

## Abstract

The preparation of a high-strength biodegradable plastic has always been the focus of academia. Here, we prepared two biodegradable composites using silane coupling-agent-modified hemp fibers (Si-HF) and unmodified hemp fibers (HF) with butylene adipate-*co*-terephthalate (PBAT), respectively. We compared the differences of Si-HF/PBAT and HF/PBAT in terms of micromorphology, density, mechanical properties, thermal stability and biodegradability. The Si-HF has better interface interaction between the hemp and the PBAT matrix than the HF, which makes Si-HF/PBAT have better tensile properties. Moreover, Si-HF/PBAT has stronger tensile strength and modulus than HF/PBAT. Our results also show that the two composites have good biodegradability. This study provides an important reference for the subsequent development and utilization of hemp fibers.

## 1. Introduction

In the past hundred years, the invention and development of synthetic plastics have greatly promoted the progress of society and technology, and also brought great convenience to people’s work and life. From drinking containers to automation components, these materials have increasingly replaced glass and metal materials [1,2]. Although polymer science has gradually matured, many challenges still existed, including issues of sustainable development and environmental pollution [3]. In order to limit and improve the “white pollution” caused by the plastics industry, countries around the world have issued increasingly stringent “plastic limit orders” [4,5,6]. As people paid more and more attention to green chemical processing, more attention was paid to the development and application of polymer materials with better environmental affinity [7,8]. Therefore, how to develop a biodegradable-polymer plastic that is good for the environment was an urgent problem to be solved.

PBAT is a thermoplastic biodegradable plastic and a copolymer of butylene adipate and butylene terephthalate [9]. However, its low mechanical properties limit its application in packaging and agriculture. Therefore, how to increase the mechanical strength of PBAT has become the focus of attention of scientists in recent years [10]. Recent studies have shown that using coffee-ground fiber to modify PBAT can increase the hydrophobicity and thermodynamic properties of PBAT composites [11]. Doping Croton lanjouwensis fiber, Malvastrum tomentosum fiber, and Trema micrantha fiber in PBAT can increase the elastic modulus of PBAT composites by 48%, 70%, and 72%, respectively [12]. The mechanical properties of PBAT composites made of Bactris gasipaes kunth fibers have been greatly improved [13]. However, despite these previous results indicating that cellulose can be used to improve the mechanical properties of PBAT, the quantity of natural fibers used in such applications is still insignificant. There are some fibers that have not yet been reported but have potential applications, such as hemp fiber from China.

Hemp has a history of thousands of years in China. The average area of hemp planted in China is 30,000–50,000 hectares, accounting for 1/3–1/2 of the world’s total planting area [14]. The fiber content of hemp is about 52% [15]. Therefore, the full utilization of hemp fiber has far-reaching significance for improving the added value of hemp. More importantly, hemp fiber is abundant, sustainable, renewable, and biodegradable [16,17,18]. Compared with other natural plant fibers such as jujube palm, jute, and flax, hemp fiber has some excellent mechanical properties (mainly the tensile strength and tensile modulus at break), and only some have been mentioned [19,20]. These fibers have a good impact on the environment, especially when compared with traditional fibers such as glass [21,22,23]. These attractive properties have led to an increase in the use of these fibers as reinforcements for composite materials. However, due to the high hygroscopicity of hemp fiber and the hydrophobicity of the polymer matrix, the compatibility between the two is poor, thereby reducing the interfacial adhesion with the matrix [18]. Therefore, the use of hemp fiber to improve the mechanical properties of PBAT composites is promising.

In the present work, we study the micromorphology, density, and mechanical properties of hemp fiber/PBAT and silane coupling-agent-modified hemp fiber/PBAT composites with different fiber additions, and the hemp-fiber reinforcement based on the Cox shear-lag model is obtained. The PBAT stretch model and the introduction of fiber-compressibility factor improve the model. Fitting the two models and the experimental data shows the effect of the effectiveness and interface performance in the cell.

## 2. Materials and Methods

### 2.1. Materials

Hemp fiber was provided by Heilongjiang Hagong Wisdom Hemp Technology Co., Ltd. (Harbin, China). Silane coupling agent KH-560 was purchased from Nantong Runfeng Petrochemical Co., Ltd. (Nantong, China). Poly(butylene adipate-*co*-terephthalate) pellets (PBAT) with density of 1.16–1.30 g/cm^3^, melting point of 110–120 °C, and transparency of 82%, supplied by Xinjiang Blue Ridge Tunhe Chemical Industry Joint Stock Co., Ltd. (Changji, China), was used as base matrix. PBAT consisted of 50 mol% of butanediol (Beijing, China), 22 mol% of adipic acid (Nanjing, China), and 28 mol% of terephthalic acid (Nanjing, China).

### 2.2. Surface Modification of Hemp Fibers

The hemp fibers were first surface-treated by alkylation: the hemp fibers were soaked in 10% NaOH solution for 1 h. After the treatment was completed, the sodium hydroxide solution was poured into the waste liquid pool, and then the hemp fiber was washed with distilled water until the cleaning solution was neutral, and the hemp fiber was taken out dry at 80 °C in an oven for later use. In a 95% ethanol aqueous solution, the pH value was adjusted to 4.5 with acetic acid, KH-560 silane coupling agent was added while stirring, and hydrolyzed for 5 min, then the alkali-treated hemp fiber was soaked for 2 h, cleaned with ethanol after treatment, and dried for later use.

### 2.3. Preparation of PBAT/HF Composite

The preparation process of the composite material was as follows: (1) Raw materials were dried in an oven—the untreated hemp fiber (HF), the silane coupling-agent-modified hemp fiber (Si-HF) and PBAT particles were dried in a constant-temperature drying oven at 80 °C for 4 h to meet the drying requirements of the subsequent research. (2) Open refining and blending—before starting the preparation, the temperature of the front roll and the back roll of the double-roll mill were increased to 150 °C and 145 °C, respectively, and preheated for 1.5 h. According to the mass fraction of hemp fibers as 3%, 5%, 10%, and 20% (3) Hot pressing. In this work, the temperature and molding pressure of the plate vulcanizer were set to 145 °C and 10 MPa, respectively. A certain quality of the molten material was weighed after the refining, and the molten material was placed in a hot-pressing mold, in which the two sides of the mold were padded with PTFE film to ensure the surface finish of the sheet after hot pressing and facilitate demolding. It was then exhausted 15 times after preheating, and then the pressure was held for 5–6 min for hot-press molding, and then cold water was used to cool it down in order to obtain the composite sheet.

### 2.4. Characterization

#### 2.4.1. Morphology

Morphology of the composites was analyzed using scanning electron microscope (Hitachi, Model S-3400N, Tokyo, Japan). The samples for the SEM analysis were cryogenically broken by dipping in liquid nitrogen, and the fracture surface was exposed to gold coating.

#### 2.4.2. Density

The density of the samples was measured using an Alfa Mirage electronic densimeter MDS 300 (Alfa Mirage, Osaka, Japan) by immersion at room temperature in distilled water following the ASTM D792 standard.

#### 2.4.3. Mechanical Properties

The tensile properties of the samples were determined using a universal-testing machine (Instron, Norwood, MA, USA) with a load cell of 10 kN, at a cross-head speed of 50 mm/min. The dumbbell-shaped samples with dimensions 75 × 5 × 2 mm^3^ were used for tensile testing according to ISO 527 standards. The samples for testing were injection-molded using an injection-molding machine (Haitian, Zhejiang, China). The gauge length between the jaws was set to 55 mm at the start of testing.

#### 2.4.4. Thermal Stability

Approximately 5 mg of dried PBAT and composite samples were placed in a TG crucible furnace (Netzsch, Selb, Germany) for thermogravimetric analysis. In the experiment, the temperature-sweep interval was set to 30~550 °C, the nitrogen purge flow rate was 20 mL/min, and the heating rate was 10 °C/min.

### 2.5. Tensile Model of Hemp-Fiber-Reinforced PBAT Composite

The most commonly used model for predicting the tensile strength of short-fiber-reinforced composites was the Cox shear-lag model proposed by [24] based on the Weibull distribution principle. The Cox shear-lag model was:

(1) $$\sigma_{c}=\eta_{LE}\sigma_{f}V_{f}+\sigma_{m}\left(1-V_{f}\right)$$

Among them, $\eta_{LE}$ is the effective factor of fiber length, and Cox also gives its calculation formula (Equations (2) and (3)):

(2) $$\eta_{LE}=1-\frac{\tanh\cdot \left(\beta L/2\right)}{\beta L/2}$$

(3) $$\beta =\sqrt{\frac{2G_{m}}{E_{f}r_{f}^{2}\ln\left(R/r_{f}\right)}}$$

Among them, *L* is the fiber length, $r_{f}$ is the radius of the fiber, $G_{m}$ is the shear modulus of the matrix, *R* is half the distance between the fibers, *R* can be obtained by Equation (4), and *G* can be obtained by Equation (5):

(4) $$R=\frac{r_{f}}{2}\times \sqrt{\frac{\pi }{V_{f}}}$$

(5) $$G_{m}=\frac{E_{m}}{2\left(1+v_{m}\right)}$$

On the basis of the Cox shear-lag model, Fukuda et al. [25] considered the influence of fiber orientation, introduced a fiber-orientation-distribution factor, and revised the tensile-strength-prediction model of short-fiber-reinforced polymer-matrix composites. He rewrote Equation (1) into the form shown in Equation (6), and calculated the fiber-orientation-distribution factor using mathematical derivation methods.

(6) $$\sigma_{c}=C_{0}\eta_{LE}\sigma_{f}V_{f}+\sigma_{m}\left(1-V_{f}\right)$$

(7) $$C_{0}=\int_{0}^{\frac{\pi }{2}}g\left(\theta \right)\cos\theta d\theta \int_{0}^{\theta_{0}}g\left(\theta \right)\cos^{3}\theta d\theta \times \int_{0}^{\theta_{0}}\left(1-\frac{\beta }{\cos\theta }\right)g\theta d$$

Among them, when the fibers are randomly distributed in the two-dimensional plane, $g\left(\theta \right)=\frac{\pi }{2}$; when the fibers are randomly distributed in the three-dimensional plane, $g\left(\theta \right)=\sin\theta$. In fact, for the fiber-orientation factor $C_{0}$, different composite material-molding processes can obtain different values. The more obvious the orientation of the fiber, the greater the value of the orientation factor $C_{0}$. In this paper, during the preparation of the composite material, the fibers are smelted by a double-roll mill and mixed with the PBAT matrix. Hemp fibers are randomly distributed in the PBAT matrix. Therefore, the value of $C_{0}$ is 0.270 by Equation (7).

Since the diameter of the fiber will affect the performance of the fiber and the properties of the interface layer between the fiber and the polymer matrix, and the diameter of the fiber will change before and after the modification, it is necessary to measure the diameter of the fiber. In this chapter, the average diameter of the fibers is used for calculation; 10 fibers are randomly selected, the diameter of the fibers are tested using a stereomicroscope, and the average value is taken.

### 2.6. Biodegradability Test

The conventional soil-burial method was used to test the biodegradability of the hemp-fiber/PBAT-composite material [26]. First, the hemp-fiber-reinforced PBAT-composite material to be tested was pressed into a 0.5 mm-thick sheet by a plate vulcanizer, then cut into a 20 mm × 20 mm × 0.5 mm sheet, and dried to a constant weight in an oven at 80 °C. The sample was buried to be tested 15 cm below the soil to ensure that the samples of each group were buried at the same depth, so that they degraded in a natural environment. Three samples were taken from each group every 10 days, washed with distilled water and ethanol successively, dried, weighed and the weight-loss rate was calculated for each group, and the biodegradability of the composite materials of each group was comprehensively evaluated through the measured weight-loss rate.

## 3. Results and Discussion

### 3.1. Morphology

**Figure 1:**
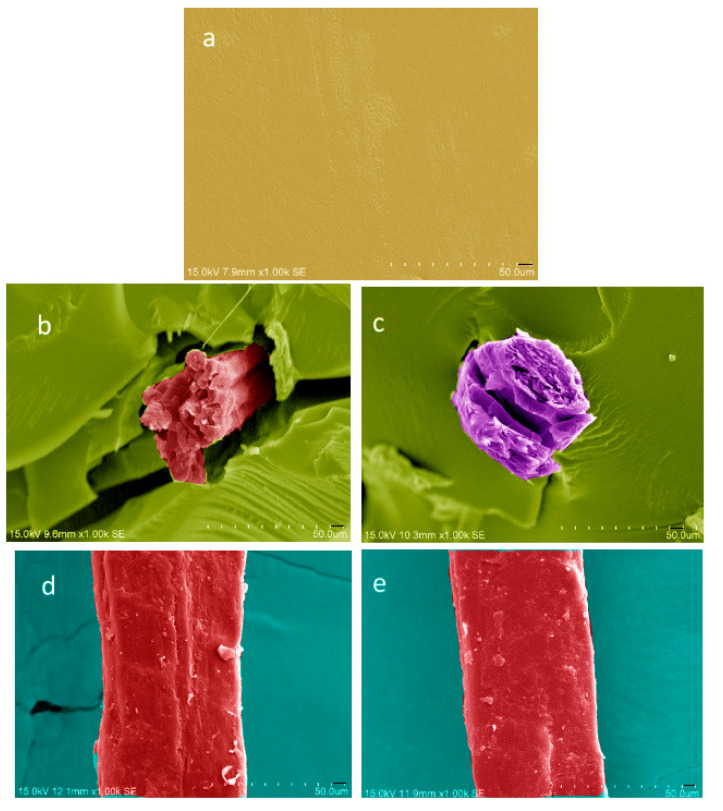
SEM micrographs of the PBAT composites: (**a**) neat PBAT, (**b**) PBAT/HF, (**c**) PBAT/Si-HF, (**d**) unmodified hemp fiber, (**e**) modified hemp fiber.

The morphology of different composites of PBAT is shown in Figure 1. Figure 1a shows the SEM micrograph of virgin PBAT. Figure 1b,c shows the SEM micrographs of untreated and silane-coupling-treated hemp-fiber-reinforced composites, respectively. Note that both untreated and silane-treated hemp fibers were pulled out from the PBAT matrix. Figure 1d,e shows the SEM micrographs of untreated and treated hemp fibers. The diameter of the fiber was reduced for the treated fibers. This was due to the removal of pellicle layers of membrane around the fiber from the fiber surface as a result of the NaOH treatment. This layer may contain pectin, lignin, and other impurities. After the treatment, the surface become free from pectin and lignin and was coated with silane. Interestingly, unlike the unmodified fiber, the surface of the modified hemp fiber was smooth, probably due to the deposition of the silane layer over the surface. We also drew a structural chemical-effect-flow diagram (Figure S1).

### 3.2. Density

**Figure 2:**
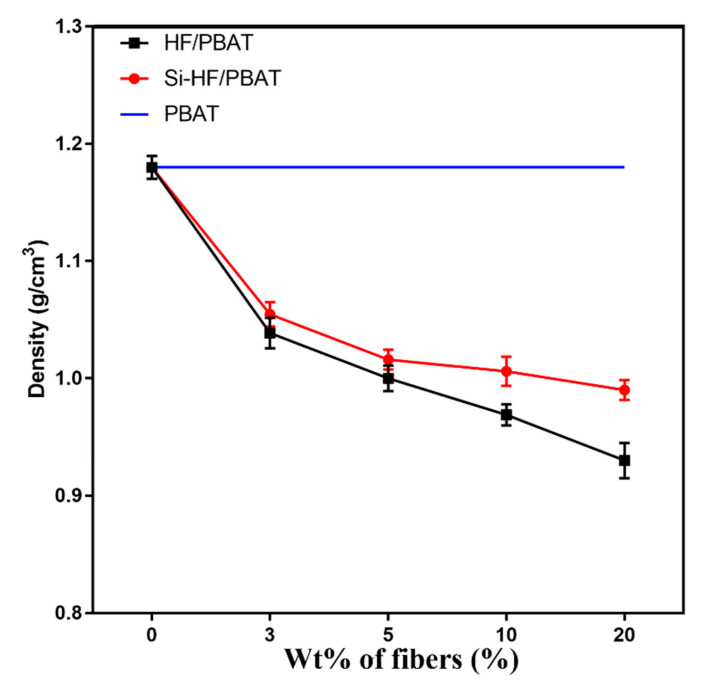
Density change of PBAT composites with hemp-fiber content.

It is well-known that density plays a crucial role in determining the characteristics of lightweight composite materials. Here, we evaluate the density of virgin PBAT and its composites (Figure 2). For many applications, lightweight composites are preferred over conventional composites due to their weight savings. Both hemp- and Si-hemp-reinforced composites showed a decrease in density as the concentration of the filler was increased. Surprisingly, the density of the composites was much lower than that of neat PBAT. It was expected not to have too much variation in densities of the samples when the fiber was added, as the density of PBAT (1.16 g/cm^3^) and density of hemp (unmodified 1.25 g/cm^3^, silane-modified 1.17 g/cm^3^) were very close to each other.

### 3.3. Tensile Properties

**Figure 3:**
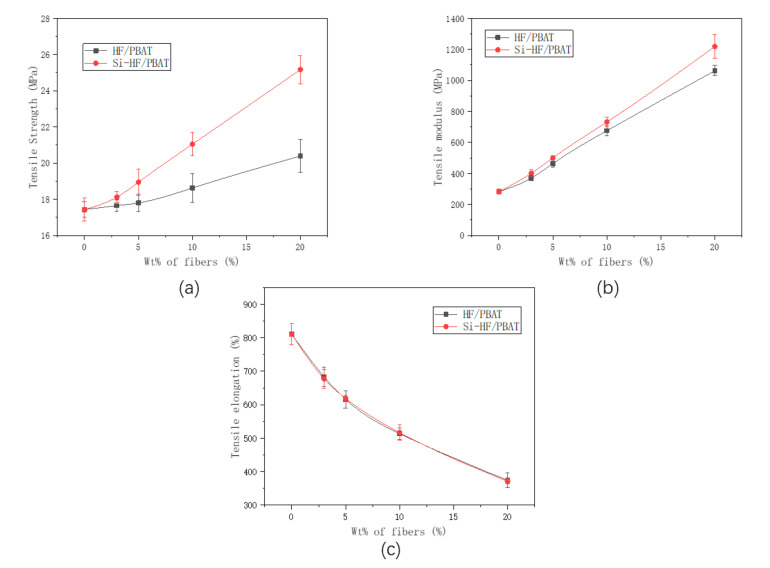
Tensile properties of neat PBAT and its composites (**a**) Tensile strength of hemp and Si-hemp, (**b**) Tensile modulus of hemp and Si-hemp, (**c**) Tensile elongation of hemp and Si-hemp.

In order to comprehensively evaluate the tensile properties of composite materials, we measured the changes in tensile strength (TS), tensile modulus (TM), and tensile elongation (TE) (Figure 3). We can determine that the TS and TM of the composite material both increase with the increase in the concentration of unmodified hemp fiber and modified hemp fiber. The TS of the composites with modified hemp increased by ca. 45% compared to ca. 16% for those with the unmodified hemp. Similarly, the TM of composites with modified hemp increased by ca. 334% compared to ca. 277% for those with the unmodified hemp. This was due to the fact that silane functionalization improved the fiber-surface adhesion to the PBAT matrix by improving the wettability of the fiber surface and thus enhanced the stress transfer between the fiber and matrix. Furthermore, the surface modification reduced the hydrophilic nature of the fiber, making it more compatible with the hydrophobic matrix. Contrary to this, the TE decreased with the increase in concentration of filler due to the reinforcement imparted by the fiber on the PBAT matrix.

### 3.4. Stretch Model

When the Fukuda model was used for the fitting calculation, it was found that Fukuda did not consider the change in the fiber diameter after the fiber-reinforcement phase was compressed and deformed when blended with the polymer matrix. In fact, previous studies have found that the interior of plant fibers was a porous structure with holes. This structure made plant fibers deform due to external shear stress and compressive stress when they were blended with the composite matrix In order to consider the influence of the compression deformation of hemp fiber in the process of blending with the PBAT matrix, the fiber-compression coefficient *K* was introduced to the model of Formula (8), becoming the following formula:

(8) $$\sigma_{c}=KC_{0}\eta_{LE}\sigma_{f}V_{f}+\sigma_{m}\left(1-V_{f}\right)$$

When calculating the average fiber-diameter distribution, we ignored the change in fiber density during the modification of hemp fibers, and considered the compression ratio *K* (the ratio of the diameter of hemp fibers before blending to the diameter of hemp fibers after blending). The diameter and compressibility of fibers before and after blending with the PBAT matrix are shown in Table 1. The diameter and compressibility of the fiber and PBAT matrix before and after blending were shown in Table 1.

After treatment with the silane coupling agent, the diameter of Si-HF was slightly larger than that of HF (Table 1), which is due to the formation of a deposition layer on the surface of the hemp fibers after modification with the silane coupling agent. In addition, Si-HF and HF decreased in diameter after blending with PBAT (Table 1).

**Figure 4:**
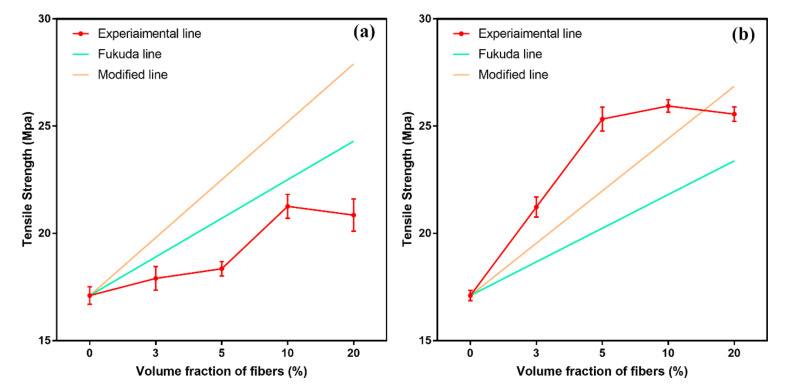
Experimental data and fitting line of tensile strength of composites (**a**) PBAT/HF; (**b**) PBAT/Si-HF.

The tensile strength and fitting curve of the two composite materials of HF/PBAT and Si-HF/PBAT are shown in Figure 4. The figure also shows the comparison between the revised curve and the Fukuda curve.

For PBAT/HF composite materials, it can be found that there is still a large deviation between the fitting curve and the experimental data, and the fitting-model data are still larger than the experimental data. However, observing the PBAT/HF experimental curve, we can find that when the fiber content in the composite material increased from 10%, the growth trend was consistent with the growth trend of the fitting model. Comparing the Fukuda curve and the modified curve, it can be found that the modified curve is more similar to the experimental data. This shows that the fiber compression during the preparation of fiber-reinforced composite materials cannot ignore the impact of fiber compression on the mechanical properties of the composite material. The compression of the fiber improved the mechanical properties of the fiber body, and at the same time promoted the penetration of the PBAT matrix into the fiber, thus leading to the enhancement of the mechanical properties of the fiber-reinforced composite material.

For PBAT/Si-HF composite materials, it can be found that similar to PBAT/HF, the Fukuda curve is quite different from the experimental data. The modified curve after introducing the compression ratio was closer to the experimental data than the Fukuda curve, but the modified curve still had a certain deviation compared with the experimental data. Considering the influence of the interface strength and the nonlinear characteristics of the experimental curve, whether it was the Fukuda model curve or the modified model proposed in this paper, only a sufficiently small interval can ensure a good enough fit.

### 3.5. Thermogravimetric Analysis

**Figure 5:**
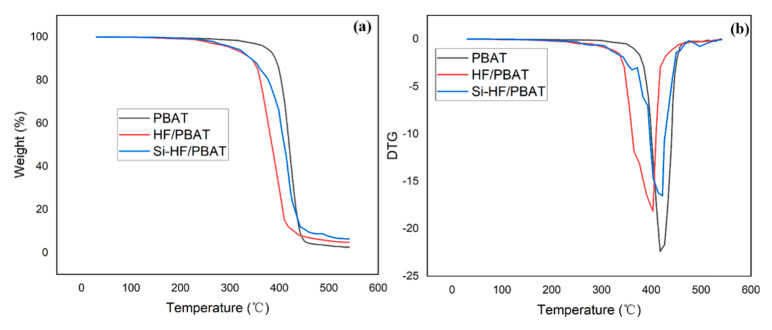
TGA (**a**) and DTG (**b**) curves for composite material. Heating rate of 10 °C/min under argon (flow rate of 100 cm^3^/min).

The thermal stability of the composite material had a greater impact on its service life and application environment. Therefore, we evaluated the thermal stability of the composite material (Figure 5). The thermal decomposition of composite materials can be divided into three processes: at 50 °C to about 300 °C, the mass of the sample slowly decreases due to the dehydration of the composite material and the decomposition of the pectin, hemicellulose, and small molecules contained in it; at 300 °C–450 °C, the mass of the sample decreases rapidly due to the decomposition of cellulose and PBAT in the composite material; above 450 °C it is due to the decomposition of the remaining substances in the composite material (Figure 5). The thermal-decomposition temperature of HF/PBAT composites was lower than that of pure PBAT, because the hemp fiber with poor thermal stability was added to the composites (Figure 5). In addition, the thermal-decomposition starting temperature and maximum thermal-decomposition-rate temperature of Si-HF/PBAT were higher than those of HF/PBAT (Figure 5). The thermal stability of Si-HF/PBAT was higher than that of HF/PBAT, which was the result of silane functionalization of hemp fiber.

### 3.6. Biodegradability Test

**Figure 6:**
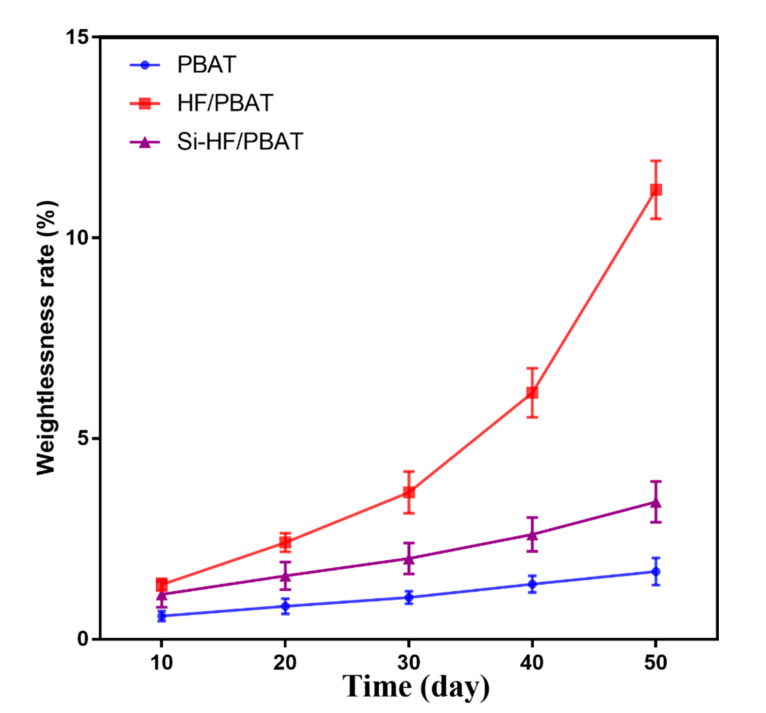
Effect of different treatment methods of hemp fiber on the biodegradability of composite materials.

Here, we tested the biodegradability of composite materials (Figure 6). When the degradation time was relatively short, the weight loss of Si-HF/PBAT and HF/PBAT composites was higher than that of pure PBAT (Figure 6). With the extension of the degradation time, the degradation rate of HF/PBAT accelerated significantly, while Si-HF/PBAT still maintained a lower weight-loss rate (Figure 6). This shows that the silane-coupling-agent modification enhances the bonding strength of the hemp fiber and the composite material, and at the same time, it also leads to a decrease in its biodegradability. Moreover, as the degradation time increases, the weight loss of HF/PBAT increases very quickly (Figure 6).

## 4. Conclusions

In this study, two biodegradable composites (Si-HF/PBAT and HF/PBAT) were prepared. HF and Si-HF can significantly reduce the density of PBAT, which indicates that Si-HF/PBAT and HF/PBAT can be used for lightweight composite applications. In addition, compared with HF, Si-HF has better interfacial interaction with PBAT. Compared with HF/PBAT, Si-HF/PBAT has better tensile strength, modulus, and thermal stability. The degradation experiments showed that both HF/PBAT and Si-HF/PBAT had good biodegradability, but the biodegradability of HF/PBAT is better than that of Si-HF/PBAT. This study provides a new perspective for the utilization of Chinese hemp resources.

## Figures and Tables

**Table 1:** Diameter and compression ratio of different fibers before and after mixing.

Fiber Type	Diameter before Blending (mm)	Diameter after Blending (mm)	Compression Ratio
HF	0.171	0.127	1.35
Si-HF	0.177	0.125	1.42

## Data Availability

The data that support the findings of this study are available from the corresponding author on reasonable request.

## Supplementary Materials

The following supporting information can be downloaded at: www.mdpi.com/article/10.3390/ma15072445/s1, Figure S1: Synthesis mechanism of hemp fiber/PBAT composites.

## References

[1] Ayre D. (2018). Technology advancing polymers and polymer composites towards sustainability: A review. Curr. Opin. Green Sustain. Chem..

[2] Pellis A., Malinconico M., Guarneri A., Gardossi L. (2021). Renewable polymers and plastics: Performance beyond the green. New Biotechnol..

[3] Chae Y., An Y.J. (2018). Current research trends on plastic pollution and ecological impacts on the soil ecosystem: A review. Environ. Pollut..

[4] Farrelly T.A., Borrelle S.B., Fuller S. (2021). The Strengths and Weaknesses of Pacific Islands Plastic Pollution Policy Frameworks. Sustainability.

[5] Prata J.C., Silva A.L.P., Costa J.P.d., Mouneyrac C., Walker T.R., Duarte A.C., Rocha-Santos T. (2019). Solutions and Integrated Strategies for the Control and Mitigation of Plastic and Microplastic Pollution. Int. J. Environ. Res. Public Health.

[6] Xanthos D., Walker T.R. (2017). International policies to reduce plastic marine pollution from single-use plastics (plastic bags and microbeads): A review. Mar. Pollut. Bull..

[7] Ali S.S., Elsamahy T., Koutra E., El-Sheekh M., Abdelkarim E.A., Zhu D., Sun J. (2021). Degradation of conventional plastic wastes in the environment: A review on current status of knowledge and future perspectives of disposal. Sci. Total Environ..

[8] Oliveira J., Belchior A., da Silva V.D., Rotter A., Petrovski Ž., Almeida P.L., Lourenço N.D., Gaudêncio S.P. (2020). Marine Environmental Plastic Pollution: Mitigation by Microorganism Degradation and Recycling Valorization. Front. Mar. Sci..

[9] Jian J., Zeng X., Huang X. (2020). An overview on synthesis, properties and applications of poly(butylene-adipate-co-terephthalate)–PBAT. Adv. Ind. Eng. Polym. Res..

[10] Lai L., Wang S., Li J., Liu P., Wu L., Wu H., Xu J., Severtson S.J., Wang W.J. (2020). Stiffening, strengthening, and toughening of biodegradable poly(butylene adipate-co-terephthalate) with a low nanoinclusion usage. Carbohydr. Polym..

[11] Moustafa H., Guizani C., Dupont C., Martin V., Jeguirim M., Dufresne A. (2017). Utilization of Torrefied Coffee Grounds as Reinforcing Agent To Produce High-Quality Biodegradable PBAT Composites for Food Packaging Applications. ACS Sustain. Chem. Eng..

[12] Ferreira F.V., Pinheiro I.F., Mariano M., Cividanes L.S., Costa J.C.M., Nascimento N.R., Kimura S.P.R., Neto J.C.M., Lona L.M.F. (2019). Environmentally friendly polymer composites based on PBAT reinforced with natural fibers from the amazon forest. Polym. Compos..

[13] Da Silva J.S.P., da Silva J.M.F., Soares B.G., Livi S., Livi S. (2017). Fully biodegradable composites based on poly(butylene adipate-co-terephthalate)/peach palm trees fiber. Compos. Part B: Eng..

[14] Salentijn E.M.J., Zhang Q., Amaducci S., Yang M., Trindade L.M. (2015). New developments in fiber hemp (*Cannabis sativa* L.) breeding. Ind. Crops Prod..

[15] Garcia-Jaldon C., Dupeyre D., Vignon M.R. (1998). Fibres from semi-retted hemp bundles by steam explosion treatment. Biomass Bioenergy.

[16] Fike J. (2017). Industrial Hemp: Renewed Opportunities for an Ancient Crop. Crit. Rev. Plant Sci..

[17] Saradava B.J., Kathwadia A.J., Goraviyala A.D., Joshi V.K. (2016). Mechanical characterization of hemp fiber reinforced polyester composites. Int. J. Sci. Dev. Res..

[18] Shahzad A. (2012). Hemp fiber and its composites—A review. J. Compos. Mater..

[19] Mohit H., Arul Mozhi Selvan V. (2018). A comprehensive review on surface modification, structure interface and bonding mechanism of plant cellulose fiber reinforced polymer based composites. Compos. Interfaces.

[20] Pereira P.H.F., de Freitas Rosa M., Cioffi M.O.H., de Carvalho Benini K.C.C., Milanese A.C., Voorwald H.J.C., Mulinari D.R. (2015). Vegetal fibers in polymeric composites: A review. Polímeros.

[21] Bledzki A.K., Gassan J. (1999). Composites reinforced with cellulose based fibres. Prog. Polym. Sci..

[22] Pandey J.K., Ahn S.H., Lee C.S., Mohanty A.K., Misra M. (2010). Recent Advances in the Application of Natural Fiber Based Composites. Macromol. Mater. Eng..

[23] Syduzzaman M., Al Faruque M.A., Bilisik K., Naebe M. (2020). Plant-Based Natural Fibre Reinforced Composites: A Review on Fabrication, Properties and Applications. Coatings.

[24] Cox H.L. (1952). The elasticity and strength of paper and other fibrous materials. Br. J. Appl. Phys..

[25] Fukuda H., Chou T.W. (1982). A probabilistic theory of the strength of short-fibre composites with variable fibre length and orientation. J. Mater. Sci..

[26] Wu S., Wang W., Zhang R., Zhai X., Hou H. (2021). Preparation and characterization of biodegradable trilayer films based on starch and polyester. Int. J. Biol. Macromol..

