# Supplementary material for: Mechanical Properties and Tensile Model of Hemp-Fiber-Reinforced Poly(butylene adipate-co-terephthalate) Composite
Source: Materials (Basel). 2022 Mar 26;15(7):2445. doi: 10.3390/ma15072445 (PMC8999462; doi:10.3390/ma15072445)
Supplement: Supplementary file 1 [file materials-15-02445-s001.zip › materials-1632278-supplementary.pdf]

materials

Supplementary

Mechanical Properties and Tensile Model of Hemp-Fiber-Reinforced Poly (Butylene Adipate-Co-Terephthalate) Composite

Deyong Zeng [1,2], Liang Zhang [1,2], Shaojin Jin [3], Youyuan Zhang [3], Cuicui Xu [3], Kai Zhou [3] and Weihong Lu [1,2,3]*

1 School of Medicine and Health, Harbin Institute of Technology, Harbin 150001, China; 18b925086@stu.hit.edu.cn (D.Z.); 19s025072@stu.hit.edu.cn (L.Z.);
2 National and Local Joint Engineering Laboratory for Synthesis, Transformation and Separation of Ex-treme Environmental Nutrients, Harbin 150001, China
3 Shandong Hagong Biological Technology Co. Ltd., Jinan 250200, China; jinsj@hitrobotgroup.com (S.J.); zhangyouyuan@hitrobotgroup.com (Y.Z.); xucc@hitrobotgroup.com (C.X.); zhouk@hitrobotgroup.com (K.Z.)
* Correspondence: lwh@hit.edu.cn; Tel.: +86-188-4587-0641

Citation: Zeng, D.; Zhang, L.; Jin, S.; Zhang, Y.; Xu, C.; Zhou, K.; Lu, W. Mechanical Properties and Tensile Model of Hemp-Fiber-Reinforced Poly (Butylene Adipate-Co-Terephthalate) Composite. *Materials* **2022**, *15*, 2445. https://doi.org/10.3390/ma15072445

Academic Editor: Debora Puglia

Received: 24 February 2022
Accepted: 25 March 2022
Published: 26 March 2022

Publisher's Note: MDPI stays neutral with regard to jurisdictional claims in published maps and institutional affiliations.

Copyright: © 2022 by the authors. Licensee MDPI, Basel, Switzerland. This article is an open access article distributed under the terms and conditions of the Creative Commons Attribution (CC BY) license (https://creativecommons.org/licenses/by/4.0/).

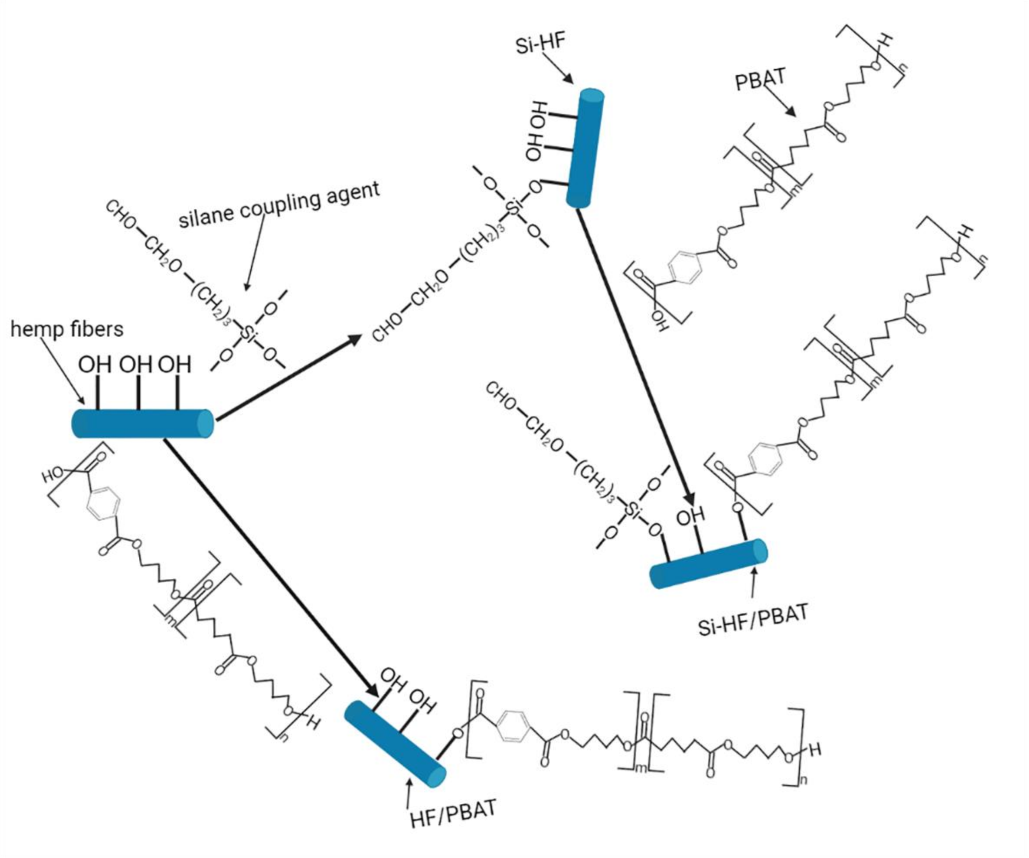

Figure S1. Synthesis mechanism of hemp fiber/PBAT composites.
